# Emerging trends and research foci of berberine on tumor from 2002 to 2021: A bibliometric article of the literature from WoSCC

**DOI:** 10.3389/fphar.2023.1122890

**Published:** 2023-03-03

**Authors:** Runzhu Yuan, Yao Tan, Ping-Hui Sun, Bo Qin, Zhen Liang

**Affiliations:** ^1^ School of Medicine, The First Affiliated Hospital of Southern University of Science and Technology, Shenzhen People’s Hospital, Shenzhen, China; ^2^ Shenzhen Aier Eye Hospital, Aier Eye Hospital, Jinan University, Shenzhen, China; ^3^ Department of Thoracic Surgery, The Second Clinical Medical College of Jinan University, Shenzhen People’s Hospital, Shenzhen, China; ^4^ Department of Geriatrics, The Second Clinical Medical College, Jinan University, Shenzhen People’s Hospital, Shenzhen, China

**Keywords:** berberine, tumor, pharmacology, bibliometric analysis, Web of Science

## Abstract

**Background:** Cancer, also known as a malignant tumor, is caused by the activation of oncogenes, which leads to the uncontrolled proliferation of cells that results in swelling. According to the World Health Organization (WHO), cancer is one of the main causes of death worldwide. The main variables limiting the efficacy of anti-tumor treatments are side effects and drug resistance. The search for natural, safe, low toxicity, and efficient chemical compounds in tumor research is essential. Berberine is a pentacyclic isoquinoline quaternary ammonium alkaloid isolated from Berberis and Coptis that has long been used in clinical settings. Studies in recent years have reported the use of berberine in cancer treatment. In this study, we performed a bibliometric analysis of berberine- and tumor-related research.

**Materials and methods:** Relevant articles from January 1, 2002, to December 31, 2021, were identified from the Web of Science Core Collection (WOSCC) of Clarivate Analytics. Microsoft Excel, CiteSpace, VOSviewer, and an online platform were used for the literary metrology analysis.

**Results:** A total of 1368 publications had unique characteristics. Publications from China were the most common (783 articles), and Y. B. Feng (from China) was the most productive author, with the highest total citations. China Medical University (Taiwan) and Sun Yat-sen University (China) were the two organizations with the largest numbers of publications (36 each). *Frontiers in Pharmacology* was the most commonly occurring journal (29 articles). The present body of research is focused on the mechanism, molecular docking, and oxidative stress of berberine in tumors.

**Conclusion:** Research on berberine and tumors was thoroughly reviewed using knowledge map and bibliometric methods. The results of this study reveal the dynamic evolution of berberine and tumor research and provide a basis for strategic planning in cancer research.

## 1 Introduction

In every nation worldwide, cancer ranks as a primary cause of mortality and a significant roadblock to increasing life expectancy (Sung et al., 2021). The mortality and incidence rates of malignant tumors have increased globally (Mullard, 2020). The World Health Organization (WHO) estimates for 2019 suggest that cancer is the third or fourth leading cause of death before the age of 70 years in 23 countries (among 183 nations in total) and the first or second leading cause in 112 countries (Sung et al., 2021). The development of cancer treatments has faced significant obstacles. While both traditional and modern approaches (surgery, radiation, chemotherapy, targeted therapy, and immunotherapy) have good efficacy, these treatment modalities also have negative consequences on patient quality of life, including the development of simultaneous resistance to multiple drugs and dermatologic toxicities (Szakacs et al., 2006; Nastiuk and Krolewski, 2016; Lacouture and Sibaud, 2018). One of the main clinical therapies for cancer is chemotherapy; however, cancer is prone to relapse and drug resistance, and most chemotherapy treatments fail; therefore, efforts to develop anti-cancer drugs are needed. Additionally, toxic side effects caused by chemotherapy also affect the quality of life of patients with cancer. Therefore, identifying anti-tumor medicines with minimal toxicity and high efficacy is crucial for the treatment of tumors.

In recent decades, scientists have conducted numerous clinical and laboratory studies on traditional Chinese medicine. Natural compounds have many medicinal properties, including multiple action targets, low toxicity, low side effects, few adverse reactions, and high safety and effectiveness. These compounds have been gradually applied to cancer treatment due to their safety, availability, accessibility, and low cost. Natural compounds have a variety of anti-cancer effects, including suppression of tumor cell growth, induction of tumor cell death, prevention of tumor spread and angiogenesis, regulation of tumor autophagy, reversal of tumor drug resistance, regulation of body immunity, and influence on tumor metabolic reprogramming (Yang et al., 2021). In addition, natural therapy can prevent many issues, increase tumor cell sensitivity to conventional treatment, reduce side effects, enhance patient quality of life, and extend patient lives to cure cancer (Sun et al., 2022a; Sun et al., 2022b). Therefore, natural medicines are receiving increasing attention. Berberine (BBR) is an isoquinoline alkaloid obtained from the Chinese herb *Coptis chinensis* and other *Berberis* species (Song et al., 2020). It is the main component of Coptidis Rhizome (CR), known as Huanglian in Chinese. Because of its pharmacological characteristics, BBR has been used as a drug to treat diseases. As a secondary metabolite of plants, it has several pharmacological characteristics (Song et al., 2020), including treatment efficacy for cardiovascular and metabolic disease (Feng et al., 2019), polycystic ovary syndrome (Zhang et al., 2021b), and non-alcoholic fatty liver disease (Koperska et al., 2022), in addition to anti-inflammatory (Kuo et al., 2004), antioxidant (Zhuang et al., 2018), and antibacterial (Li et al., 2019) properties. In recent years, BBR has also been used in the research of cancers. For example, BBR binds RXRα to suppress β-catenin signaling, leading to the inhibition of colorectal cancer proliferation (Ruan et al., 2017); BBR also regulates the HMGB1–TLR4 axis to repress the metastasis of breast cancer *in vitro* and *in vivo* (Zheng et al., 2021). Moreover, BBR exerts therapeutic actions on gastric cancer by multi-step actions such as inhibiting cell proliferation, migration, and angiogenesis (Liu et al., 2022a); BBR also combines with cisplatin to induce necroptosis and apoptosis in ovarian cancer (Liu et al., 2019). In recent years, more studies on BBR have been conducted *in vitro* and *in vivo*, which have largely proved the reproducibility and transformation potential of BBR’s anti-tumor effect in *in vitro* cell and *in vivo* animal models.

Using measurement techniques from mathematics and statistics, bibliometrics analysis assesses and forecasts the current state of science and technology by utilizing the features of the literature system as its research subject (Chen et al., 2014; Yu et al., 2020). By examining and evaluating the quantity and quality of scientific literature associated with a specific topic, bibliometric evaluation can objectively assess the state and level of development of that field (Musbahi et al., 2022). Bibliometrics assists researchers in swiftly identifying the information context in the target field, including the annual publishing trends of the literature, catalog of publishing institutes or journals, and popular research (Yin et al., 2022). The knowledge structure can be understood more methodically and intuitively using this approach, and frontiers or hotspots in a particular research area can be identified (Musbahi et al., 2022). Therefore, the present bibliometrics study aimed to investigate the role of BBR in tumors and to offer a broad perspective and roadmap for future research on BBR in pan-carcinoma treatment.

## 2 Materials and methods

### 2.1 Data source and searching strategies

**FIGURE 1:**
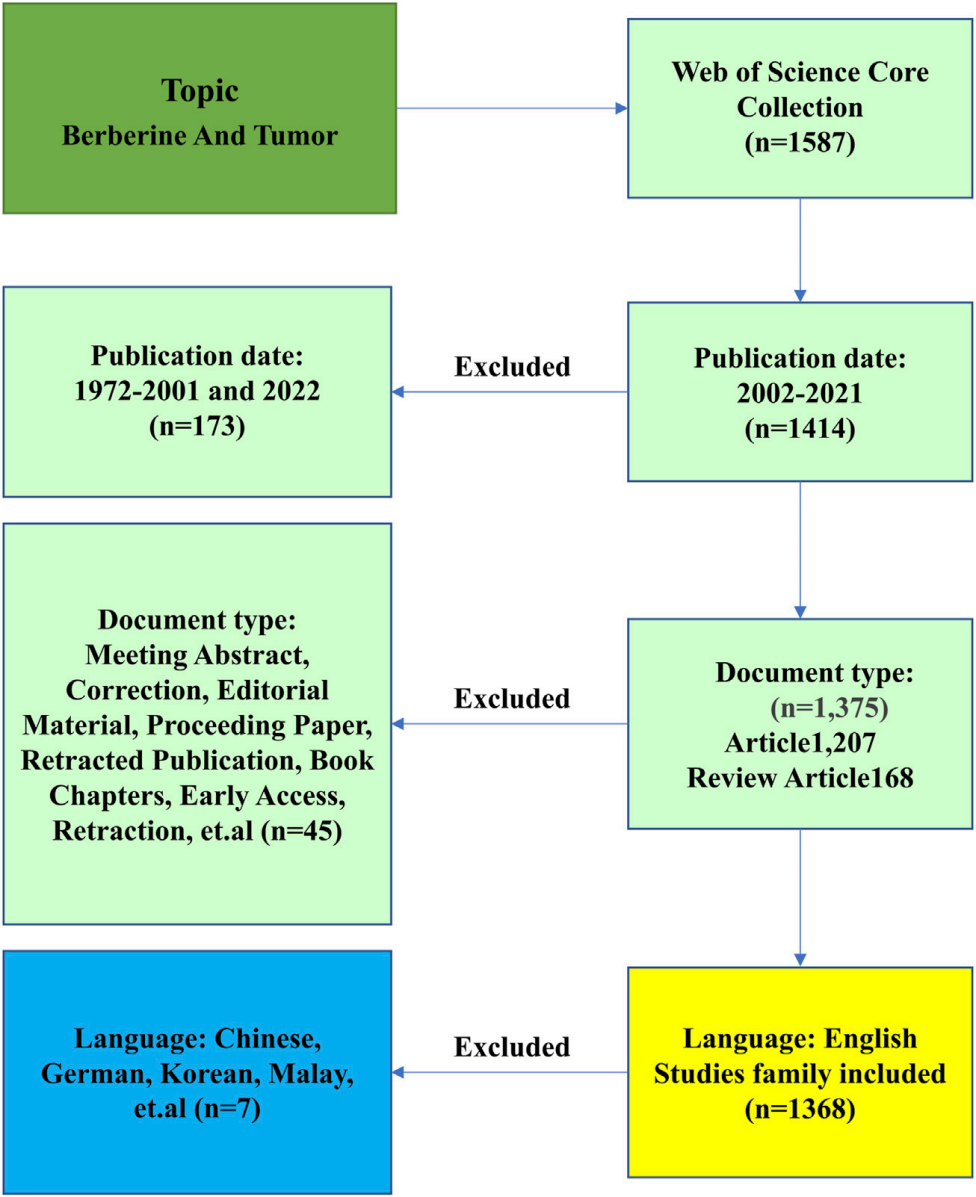
Flowchart of the screening process.

Published studies from January 1, 2002, to December 31, 2021, related to BBR and tumors were collected from the Science Citation Index Expanded (SCI-E) of the Web of Science (WoS) Core Collection (WOSCC) of Clarivate Analytics. To guarantee the reliability and accuracy of the findings, we performed pertinent pretests and improved the retrieval method. The retrieval method is illustrated in Figure 1. We used the WoS engine to search for terms related to BBR and cancer that were obtained from the Medical Subject Headings (MeSH) in PubMed. The search formula was TS = (Berberine or Umbellatine) and TS = (Tumor or Neoplasm or Tumors or Neoplasia or Neoplasias or Cancer or Cancers or “Malignant Neoplasm” or Malignancy or Malignancies or “Malignant Neoplasms” or “Neoplasm, Malignant” or “Neoplasms, Malignant” or “Benign Neoplasms” or “Benign Neoplasm” or “Neoplasms Benign” or “Neoplasm Benign”). Only publications written in English were included. The reasons for exclusion from the study were 1) no connection between BBR and tumors of any kind; 2) meeting abstract, correction, editorial material, proceeding paper, retracted publication, book chapters, early access, retraction, *etc.*; and 3) publications in a language other than English. A total of 1,368 papers were obtained.

### 2.2 Data collection

All retrieved documents were used for the bibliometric analysis. We extracted information including titles, annual publications, countries and institutes, authors, references, keywords, and scientific cooperation analysis. Data were independently extracted from a set of articles by TY and YRZ. SP-H mediated the outcomes if a disagreement arose.

### 2.3 Data analysis and visualization

We used CiteSpace (version 6.3. R3), VOSviewer (version 1.6.18), Bibliometrix (version 3.1.4), R language (version 3.6.3), and Microsoft Excel 365 for data analysis and presentation.

CiteSpace, developed by Prof. Chao-mei Chen, is a bibliometric mapping analytical tool used worldwide, most predominantly in China (Qin et al., 2022). It is a free program for analyzing, identifying, and visualizing trends and patterns in scientific literature and was chosen as the analysis target (Pan et al., 2017). In our investigation, the specifics of the CiteSpace settings were as follows: time slices of 1 year each were taken from 2002 to 2021. The links (strength: cosine; scope: inside slices) and term sources (title, abstract, author keywords, and keywords plus) were set to the default values. Items with a g-index citation or occurrence were selected for this study. Links that were not necessary were removed using Pathfinder.

VOSviewer, developed by van Eck and Waltman, creates visual network maps of scientific information, including bibliometric network analysis (van Eck and Waltman, 2010). We used VOSviewer to create network, overlay, and density visualization maps. We chose the “full counting” method for our analysis. For keywords and co-cited journals, the thresholds for the minimum numbers were set at 100 and 5, respectively. Keywords or co-cited journals are displayed as nodes in the form of a network through a visualization diagram.

Visualization of the annual publication numbers; country, institute, author, and journal rankings; cited reference bursts; and most globally and locally cited Local Cited Documents, was performed using Microsoft Excel 365. Additionally, we performed a descriptive study of writer output over time and keyword evolution in Bibliometrix (Cheng et al., 2022). Bibliometrix (https://www.bibliometrix.org) is an open-source instrument for quantitative scient-metric research. Machine-learning software was used to evaluate the distribution of each component examined in the bibliometric investigation. The variables were annual scientific production, average citations per year, most impactful journals by H-index or total citations (TC), top journals’ production over time, most relevant authors, most globally and locally cited documents, most pertinent affiliations, country-level scientific output, international collaboration network, country of origin of the corresponding author, top producing nations over time, historical direct citation network, most widely cited publications, most pertinent keywords, and keyword cluster analysis. Impact factor (IF) and partition information for the journals referred to the “Journal Citation Report (JCR) 2022.” These analytical methods offer unbiased and varied viewpoints on the function of berberine in tumor formation. The variables are presented as numbers and percentages in the descriptive study. *p*-values were not reported because no comparisons were made.

## 3 Results

### 3.1 Global publication outputs

We assessed the historical development process, current research conditions, and forecasted future trends in development by statistically analyzing the general usage of BBR pharmacological functions in the number of publications over time.

**FIGURE 2:**
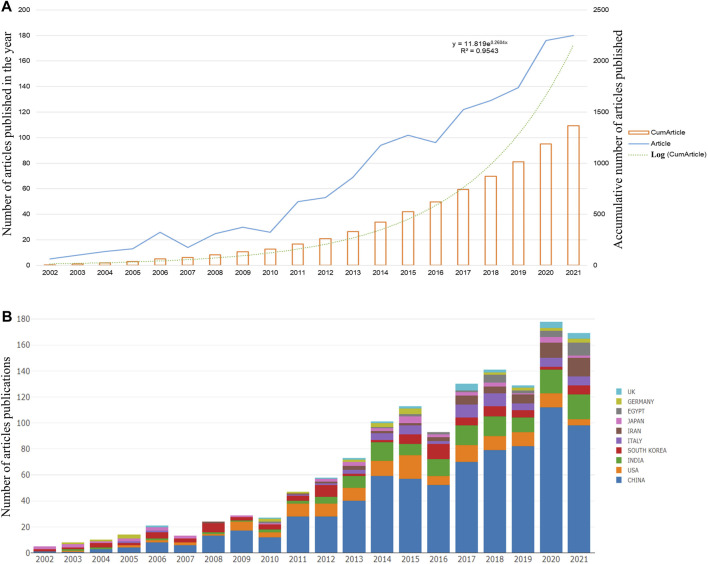
Global trends of publications about berberine and tumor. **(A)** The number of articles published each year and the accumulative number of articles from 2002 to 2021. **(B)** The annual number of papers published by nations from 2002 to 2021.

We counted the number of papers related to “berberine and tumors” from 1972 to 2021 and collected 1,587 articles for bibliographic records from the WOSCC. Ultimately, 1,368 articles were eligible for the next stage of analysis based on the exclusion criteria of publication time, document type, and language. Figure 2A shows the number of articles published each year and the cumulative number of articles published from 2002 to 2021. The overall trend has increased over the past 20 years. The greatest number of papers was published in 2021, with 180 research articles. The exponential curve equation for the rise in literature production was y = 11.819e^0.2604x^, which conforms to Price’s law. The simulation curve had a relatively strong coefficient of determination (*R* ^2^ = 0.9543), which fitted the annual literature growth trend well. Based on this curve, we predicted that the number of annual articles will steadily increase, indicating increased interest in research on BBR and tumors.

### 3.2 Distributions of countries/regions

Statistics on the research countries reporting on publications related to “berberine and tumors” showed the stages of BBR pharmacology development in each country and made comparisons easier. According to WoS, many countries have participated in research in the last 20 years. Table 1 lists the top 10 productive countries. China had the most publications (783 articles), accounting for 57.24% of the total, followed by the United States and India (142 articles each, accounting for 10.38% each, respectively), South Korea (*n* = 97), Italy (*n* = 61), Iran (*n* = 60), Japan (*n* = 42), Egypt (*n* = 30), Germany (*n* = 26), and Poland (*n* = 24).

**TABLE 1:** Top 10 countries that contributed publications on BBR and tumors.

Rank	Countries	Article count	Percentage (n/1368)	H-index	Total citations	Average citation per article
1	China	783	57.24%	70	23,603	30.14
2	United States	142	10.38%	48	6,886	48.49
3	India	142	10.38%	35	4,137	29.13
4	Republic of Korea	97	7.09%	34	3,198	32.97
5	Italy	61	4.46%	25	2,257	37.00
6	Iran	60	4.39%	22	1,800	30.00
7	Japan	42	3.07%	22	1,403	33.40
8	Egypt	30	2.19%	14	615	20.50
9	Germany	26	1.90%	16	1,117	42.96
10	Poland	24	1.75%	14	539	22.46

Publications from China ranked first in total number of citations, but the average number of citation per paper was very low, only 30.14%, compared to the top 10 countries. Although the number of articles published by Germany was limited compared to China, the average citation per article ranked second among the top 10 countries. This finding suggested that the publications were of very high quality and had certain reference values. The H-index is a mixed quantitative indicator that considers both the number of posts and the required number of citations and can be used to identify influential researchers (Hirsch, 2005). China’s researchers had the highest H-indexes, demonstrating the significant impact of their publications. After 2008, most global publications came from China. Figure 2B shows the total number of papers published worldwide from 2002 to 2021.

**FIGURE 3:**
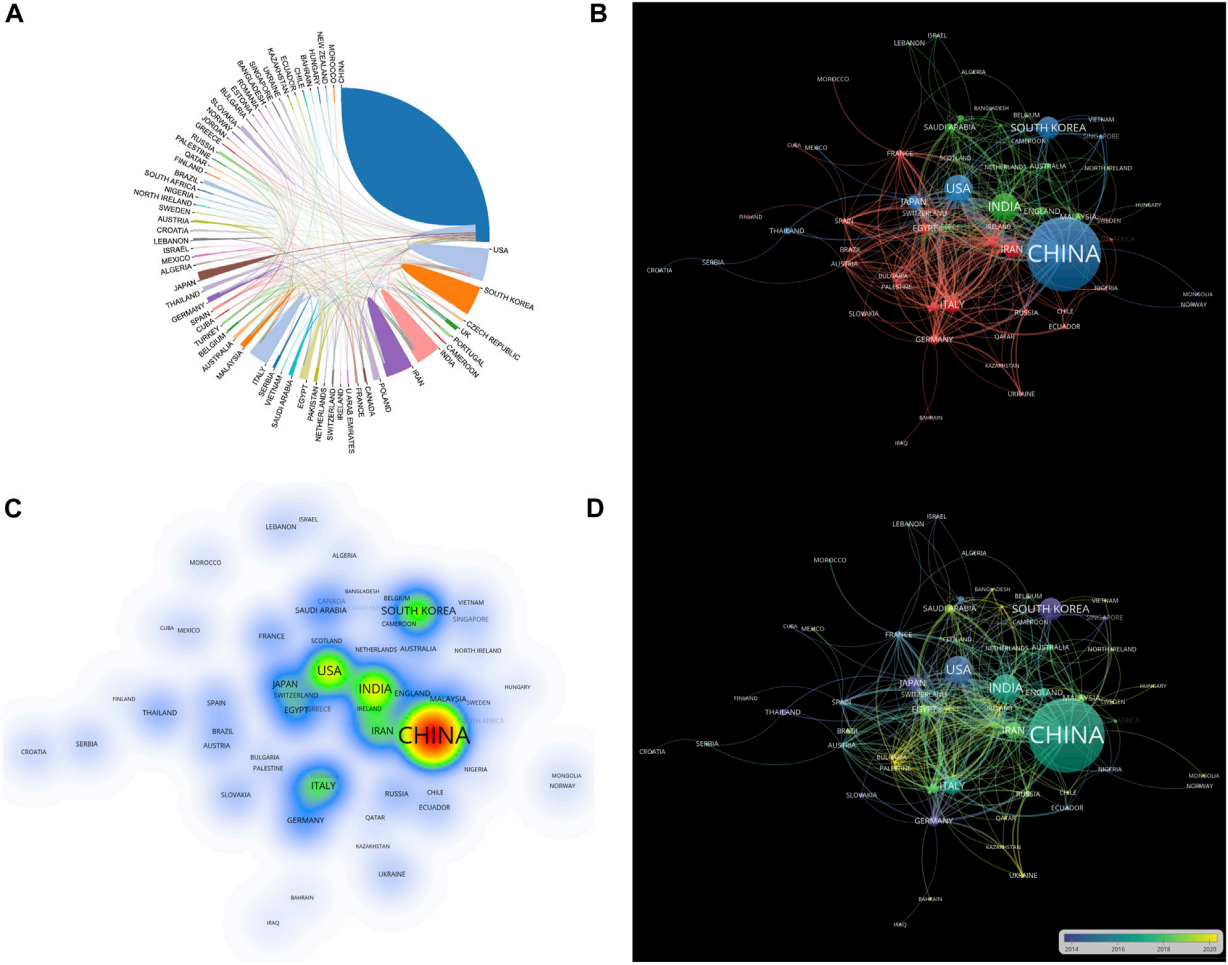
Overview of national publications. **(A)** The scientific collaborations among worldwide countries. **(B)** Visual analysis of cooperation between countries based on VOSviewer. **(C)** A density visualization map of number of publications in each nation. **(D)** Median time chart of the number of articles issued by each country.

Using an online bibliometric analysis platform, we evaluated the significance of nations in the visualization of cooperative networks. Katz and Martin, two scientists, defined scientific cooperation as the study of academics collaborating to create new scientific knowledge, known as scientific collaboration (Katz and Martin, 1997). Figure 3A shows partnerships between countries, among which articles from China showed the most aggressive cooperation with other countries; most often between China and the United States. We conducted a visual analysis using VOSviewer (Figure 3B). The circles in the map represent countries and the lines indicate the connections between them. The larger the area of the circle, the larger the contribution of these countries to this field. We found that China made significant contributions to research on BBR and cancer. A density map can be used to determine the number of publications in each nation. The lighter the color, the greater the number of publications. The density map was used to determine the number of publications in each nation (Figure 3C **)**. As shown in Figure 3C, China had the highest number of publications. Figure 3D shows that American publications were generally concentrated before 2016, whereas Chinese, Indian, Italian, and Iranian articles were concentrated after 2016.

### 3.3 Distribution of institutes

**FIGURE 4:**
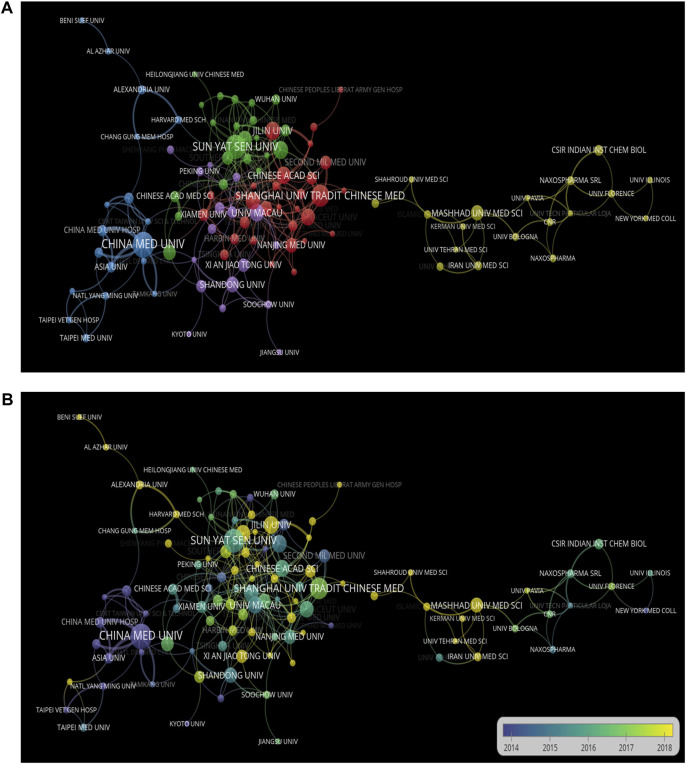
Cooperation networks between institutes based on VOSviewer. **(A)** Cooperative relationship of various institutions using network map. **(B)** Cooperative time of various institutions using network map.

The top 10 productive institutes are listed in Table 2. China Medical University (Taiwan) and Sun Yat-Sen University (China) had the most publications, with 36 articles each. China Medical University (Taiwan) showed the highest total number of citations (1,417) and H-index (21), with an average number of citations of 39.26, demonstrating a high level of acclaim for its articles. We used VOSviewer to depict the cooperation between institutes in Figure 4A. Figure 4B shows each institute’s cooperation timeline from 2002 to 2021; the node color on this map corresponds to each institute’s cooperation respective average appearing year (AAY). Jilin University and Mashhad University of Medical Sciences had relatively fresh entries compared to the nations shown in purple, such as China Medical University (Taiwan), according to the color gradient in the lower right corner.

**TABLE 2:** Top 10 institutes that contributed publications on BBR and tumors.

Rank	Institute	Country	Article count	H-index	Total number of citations	Average number of citations per article
1	China Medical University (Taiwan)	China	36	21	1,417	39.36
2	Sun Yat-Sen University	China	36	20	1,204	33.44
3	Council of Scientific Industrial Research	India	34	17	898	26.41
4	Shanghai University of Traditional Chinese Medicine	China	31	16	878	28.32
5	Chinese Academy of Sciences	China	29	18	755	26.03
6	Egyptian Knowledge Bank	Egyptian	28	14	606	21.64
7	University of Hong Kong	China	24	18	1,401	58.38
8	Guangzhou University of Chinese Medicine	China	24	14	587	24.46
9	Jilin University	China	24	14	562	23.42
10	Jinan University	China	24	14	484	20.17

### 3.4 Authors

The top 10 authors in terms of the number of articles in the past 20 years are listed in Table 3. Y. B. Feng of the University of Hong Kong, was the most prolific author (21 articles) and also received the highest number of total citations (1,281) and had an H-index of 17, demonstrating his significant contributions to the field. O Paolo from Naxospharma published 17 articles, while L Paolo and K Gopinatha Suresh (Indian Institution of Chemical Biology) both had H-index values of 12.

**TABLE 3:** Top 10 authors that contributed publications on BBR and tumors.

Rank	Author	Article count	H-index	Country	Total number of citations	Average number of citations per article	Institution
1	Feng, Y. B	21	17	China	1,281	61.00	University of Hong Kong
2	Paolo. L	17	12	Italy	879	51.71	Naxospharma
3	Gopinatha Suresh. K	16	12	Indian	510	31.88	Indian Institute of Chemical Biology
4	Tan, H.-Y	13	9	China	408	31.38	Hong Kong Baptist University
5	Wang, Y. T	11	11	China	1,094	99.45	University of Macau
6	Zheng, X	11	9	China	275	25.00	Jilin University
7	Bhupendra M. M	10	8	Republic of Korea	129	12.90	Konkuk University
8	Shao, D	10	9	China	256	25.60	Jilin University
9	Chen, L	10	8	China	297	29.70	Jilin University
10	Doo Hwan. K	10	8	Republic of Korea	129	12.90	Konkuk University

### 3.5 Journals and co-cited journals

**FIGURE 5:**
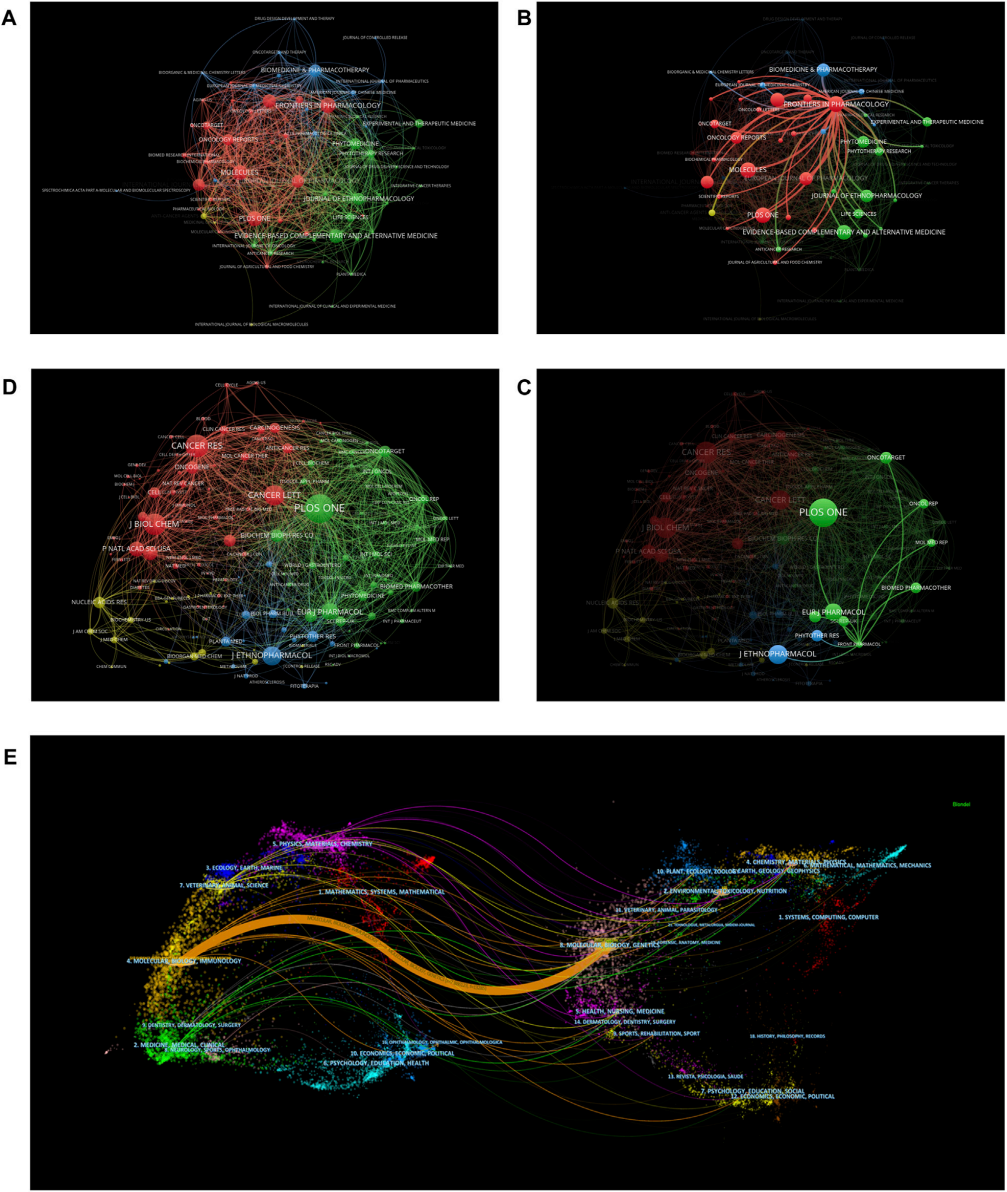
The co-cited map of journals. **(A,B)** The map of journals that published papers about berberine and tumor. **(C,D)** The map of the cited journals based on Vosviewer. **(E)** The dual-map overlay of journals.

The top 10 journals according to the number of publications are shown in Table 4 and Figure 5. The top 10 journals published 252 papers, or 18.42% of all the included publications. *Frontiers in Pharmacology* (IF = 5.9879) was the most popular journal with 29 articles, followed by *Molecules* (IF = 4.9269) with 28 articles. *PLOS One* (IF = 3.7521) was the most frequently cited journal (1,388 citations). The distribution of journals across fields, evolution of citation trajectories, and drift of scientific research centers can be displayed using the dual-map overlay of journals (Chen and Leydesdorff, 2014; Zhang et al., 2021a). In our dual-map overlay analysis (Figure 5E), the left side shows the distribution of the journals where the citing documents were located, representing the main disciplines of science mapping, while the right side shows the distribution of the journals corresponding to the cited literature, representing which disciplines were mainly cited by science mapping. The results showed that research related to BBR and tumors was mainly associated with the areas of molecular/biology/immunology and was often cited by molecular/biology/genetics researchers.

**TABLE 4:** Top 10 journals and co-cited journals that published articles on BBR and tumors.

Rank	Journal	Count	Total number of citations	Average citation per article	IF and JCR division (2021)	Co-cited journal	Total number of co-citations
1	*Frontiers in Pharmacology*	29	835	28.79	5.9879, Q1	PLOS One	1394
2	*Molecules*	28	690	24.64	4.9269, Q2	*Cancer Research*	1101
3	*PLOS One*	28	1,388	49.57	3.7521, Q2	*Journal of Biological Chemistry*	1037
4	*Evidence-Based Complementary and Alternative Medicine*	27	415	15.37	2.6498, Q3	*Cancer Letters*	973
5	*Molecular Medicine Reports*	25	484	19.36	3.4232, Q3	*Journal of Ethnopharmacology*	910
6	*Biomedicine and Pharmacotherapy*	24	954	39.75	7.4194, Q1	*European Journal of Pharmacology*	827
7	*European Journal of Pharmacology*	24	814	33.92	5.1948, Q2	*Proceedings of the National Academy of Sciences of the United States of America*	717
8	*International Journal of Molecular Sciences*	23	453	19.70	6.2082, Q1/Q2	*Nature*	687
9	*Journal of Ethnopharmacology*	23	1,234	53.65	5.1948, Q1/Q2	*Oncogene*	598
10	*Oncology Reports*	21	483	23.00	4.1361, Q3	*Biochemical and Biophysical Research Communications*	594

### 3.6 Co-cited references

**FIGURE 6:**
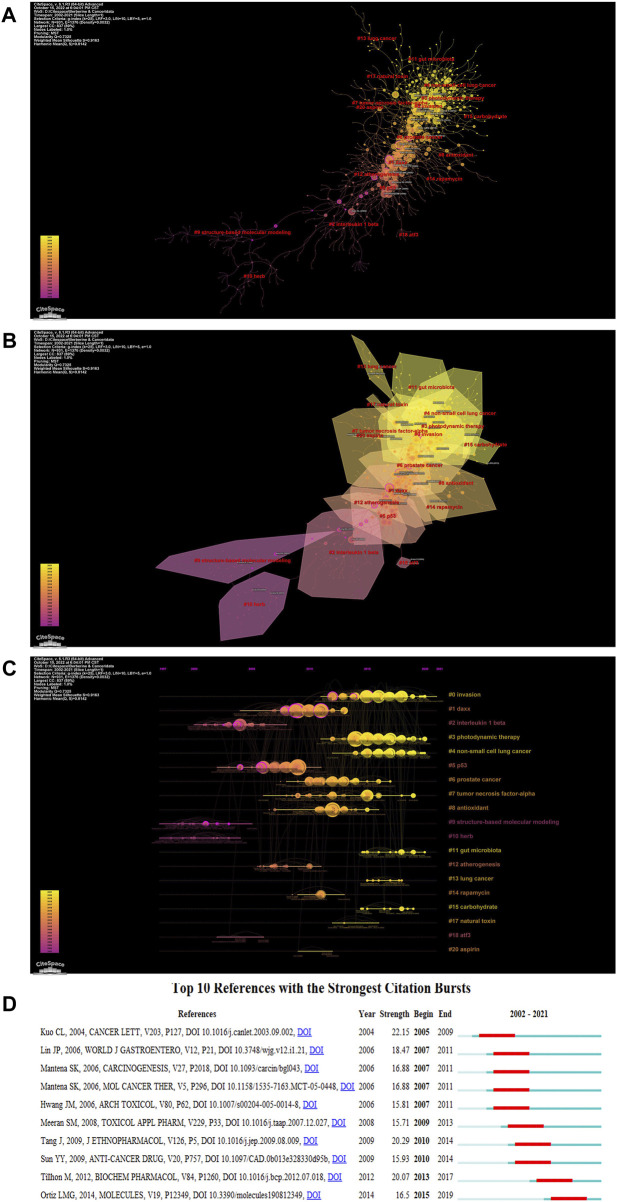
Analysis of co-cited references. **(A)** Visualized network diagram of co-cited documents. **(B)** Cluster Analysis of the co-cited articles. **(C)** Timeline view of the co-cited articles. **(D)** The top 10 references with the strongest citation bursts.

We quantified the knowledge and development of research on a particular topic by evaluating the significant nodes and clusters in the co-cited reference network (Yin et al., 2022). We used CiteSpace to create a timeline-visualized diagram network map of the co-cited articles, which was divided into 19 clusters (Table 5). The co-cited reference network is shown in Figure 6A, in which the node size is the proportion of the research frequency, color is the proportion of the time slice, and the connections are represented by lines. We further conducted cluster analysis on these co-cited articles based on the keywords in those articles (Figure 6B). By removing phrases from the titles of the cited publications, we identified the main research hotspots. Figure 6C shows a timeline of references in the field of BBR and tumors, as determined by CiteSpace. We observed a shift in the focus of research over time. The development of clusters 2 (interleukin 1 beta), 9 (structure-based molecular modeling), and 10 (herb) occurred the earliest, indicating that early research focused more on BBR structure and pharmacology. Clusters 1 (Daxx), 6 (prostate cancer), and 7 (tumor necrosis factor-alpha) occurred between 2007 and 2011, indicating a focus on the anti-cancer molecular mechanism of BBR. Clusters 15 (carbohydrate), 3 (photodynamic therapy), 4 (non-small cell lung cancer), and 11 (gut microbiota) are current research hotspots. Figure 6D shows the top 10 references with the strongest citation bursts, highlighting their significance in the fields of BBR and tumor-related research. In addition, we list the total citations of articles and local citations of articles in Tables 6 and Table 7, respectively.

**TABLE 5:** Major clusters of co-cited references.

Cluster ID	Size	Silhouette	Mean (year)	Top terms (LSI)	Top terms (LLR)	Terms (MI)
0	96	0.842	2015	Berberine; ERK1/2; and glioma	Invasion; migration; and glioma	Triptolide; RT-R breast cancer cells; and chemoprotective agents
1	86	0.884	2008	Breast cancer; signaling pathways; and leukemia	Daxx; side population; and invasiveness	Daxx; side population; and invasiveness
2	85	0.935	2003	Berberine; thermodynamics; and non-cooperative binding	Interleukin-1 beta; ARPE-19 cells; and interleukin-8	Berberine; berberine interleukin-1 beta; and cooperative binding
3	78	0.887	2016	Berberine; carrier-free; and gram-scale	Photodynamic therapy; and epithelial–mesenchymal transition	Transforming growth factor; rat; and reverse pharmacophore mapping
4	70	0.907	2017	Berberine; doxorubicin; and neurotoxicity	Non-small-cell lung cancer; liquid crystalline nanoparticles; and neurotoxicity	Demethyleneberberine; modern science; and top2b
5	69	0.95	2006	Berberine; apoptosis; and ATF3	P53; apoptosis; and *Coscinium fenstratum*	Human liver microsomes; anti-metastasis; and *Tinospora cordifolia*
6	59	0.878	2012	Berberine; IL-8; and adhesion	Prostate cancer; evodiamine; and epigenetics	Interleukin-8; traditional Chinese medicine; and gemcitabine
7	51	0.914	2012	Tumor necrosis factor-alpha; brain inflammation; and interleukin-1 beta	TNF-α; lipopolysaccharide; and NF-κB	Berberine intestinal mucosal barrier; interleukin-1; and interleukin-6
8	45	0.903	2011	Cervical cancer; gastrointestinal disorders; and antitumor	Antioxidant; piperazine; and cervical cancer	RNA triplex; g-quadruplexes; and catenin
9	45	0.971	2000	Human topoisomerase; anti-cancer drugs; and structure-based molecular modeling	Structure-based molecular modeling; interleukin-12; p38 MAPK; and anti-cancer drugs	Berberine; apoptosis; and structure-based molecular modeling
10	43	0.994	2000	Anticancer activity; anticancer effects; and colorectal cancer	Herb; colon 26/clone 20 cells; and pancreatic cancer	Berberine; apoptosis; and colon 26/clone 20 cells
11	30	0.97	2017	Gut microbiota; pharmacokinetic study; and amino acid	Gut microbiota; atherosclerosis; and metabolomics	Metabolomics; fecal metabolites; and functional nano-vector
12	22	0.942	2007	Berberine; barrier function; plant alkaloid; and lipid mediators	Atherogenesis; anti-microbial; and acute intestinal symptoms	Berberine; atherogenesis; and anti-microbial
13	16	0.997	2017	Lung cancer; inhalable nanocomposites; and drug nanosuspension	Lung cancer; lactoferrin targeting; and drug nanosuspension	Berberine; apoptosis; and lactoferrin targeting
14	13	0.957	2010	Replication stress; H_2_AX phosphorylation; DNA damage; and ribosomal protein S6 phosphorylation	Rapamycin; replication stress; and salicylate	Berberine; salicylate; and ribosomal protein S6 phosphorylation
15	10	0.995	2016	Alkylated; HeLa cell; and carbohydrate	Carbohydrate; alkylated; and anticancer	Berberine; carbohydrate; and alkylated
17	8	0.983	2014	Kainic acid; status epilepticus; and oxidative stress	Natural toxin; chronic unpredictable mild stress; and temporal lobe epilepsy	Berberine; natural toxin; and chronic unpredictable mild stress
18	7	0.998	2004	Berberine; ATF3; and apoptosis	ATF3; nag-1; p53; and apoptosis	Berberine; apoptosis; cancer; and breast cancer
20	4	0.99	2011	AMPK; aspirin; and berberine	Aspirin; metformin; AMPK; and resveratrol	Berberine; apoptosis; and cancer

**TABLE 6:** Total article citations.

Rank	Title	DOI	Total number of citations	TC per year	Normalized TC
1	Regulation of survival, proliferation, invasion, angiogenesis, and metastasis of tumor cells through modulation of inflammatory pathways by nutraceuticals	10.1007/s10555-010-9235-2	570	43.85	7.15
2	The anti-inflammatory potential of berberine *in vitro* and *in vivo*	10.1016/j.canlet.2003.09.002	515	27.11	5.91
3	Pharmacological and therapeutic effects of *Berberis vulgaris* and its active constituent, berberine	10.1002/ptr.2399	430	28.67	5.26
4	Berberine and Coptidis rhizoma as novel antineoplastic agents: A review of traditional use and biomedical investigations	10.1016/j.jep.2009.08.009	394	28.14	5.20
5	A systematic review of the anticancer properties of berberine, a natural product from Chinese herbs	10.1097/CAD.0b013e328330d95b	309	22.07	4.08
6	Berberine, a natural product, induces G1-phase cell cycle arrest and caspase-3-dependent apoptosis in human prostate carcinoma cells	10.1158/1535-7163.MCT-05-0448	285	16.76	3.59
7	Berberine: New perspectives for old remedies	10.1016/j.bcp.2012.07.018	284	25.82	6.05
8	Anti-cancer natural products isolated from Chinese medicinal herbs	10.1186/1749-8546-6-27	264	22.00	5.03
9	Targeting apoptosis pathways in cancer by Chinese medicine	10.1016/j.canlet.2010.07.015	220	22.00	5.41
10	Cancer prevention and therapy through the modulation of the tumor microenvironment	10.1016/j.semcancer.2015.02.007	212	26.50	6.11

**TABLE 7:** The local citations of articles.

Rank	Document	DOI	Year	Local Citations	Global Citations	LC/GC Ratio (%)	Normalized Local Citations	Normalized Global Citations
1	The anti-inflammatory potential of berberine *in vitro* and *in vivo*	10.1016/j.canlet.2003.09.002	2004	155	515	30.10	6.25	5.91
2	Berberine, a natural product, induces G1-phase cell cycle arrest and caspase-3-dependent apoptosis in human prostate carcinoma cells	10.1158/1535-7163.MCT-05-0448	2006	124	285	43.51	4.61	3.59
3	A systematic review of the anticancer properties of berberine, a natural product from Chinese herbs	10.1097/CAD.0b013e328330d95b	2009	121	309	39.16	4.63	4.08
4	Berberine and Coptidis rhizoma as novel antineoplastic agents: a review of traditional use and biomedical investigations	10.1016/j.jep.2009.08.009	2009	115	394	29.19	4.40	5.20
5	Berberine: new perspectives for old remedies	10.1016/j.bcp.2012.07.018	2012	103	284	36.27	7.57	6.05
6	Berberine induces apoptosis through a mitochondria/caspase’s pathway in human hepatoma cells	10.1007/s00204-005-0014-8	2006	86	170	50.59	3.20	2.14
7	Berberine induces autophagic cell death and mitochondrial apoptosis in liver cancer cells: the cellular mechanism	10.1002/jcb.22869	2010	85	200	42.50	5.44	2.51
8	Berberine inhibits growth, induces G1 arrest and apoptosis in human epidermoid carcinoma A431 cells by regulating Cdki-Cdk-cyclin cascade, disruption of mitochondrial membrane potential and cleavage of caspase 3 and PARP	10.1093/carcin/bgl043	2006	83	161	51.55	3.09	2.03
9	Pharmacological and therapeutic effects of Berberis vulgaris and its active constituent, berberine	10.1002/ptr.2399	2008	83	430	19.30	3.31	5.26
10	Berberine, an epiphany against cancer	10.3390/molecules190812349	2014	79	165	47.88	10.53	4.19

### 3.7 Analysis of keywords

**FIGURE 7:**
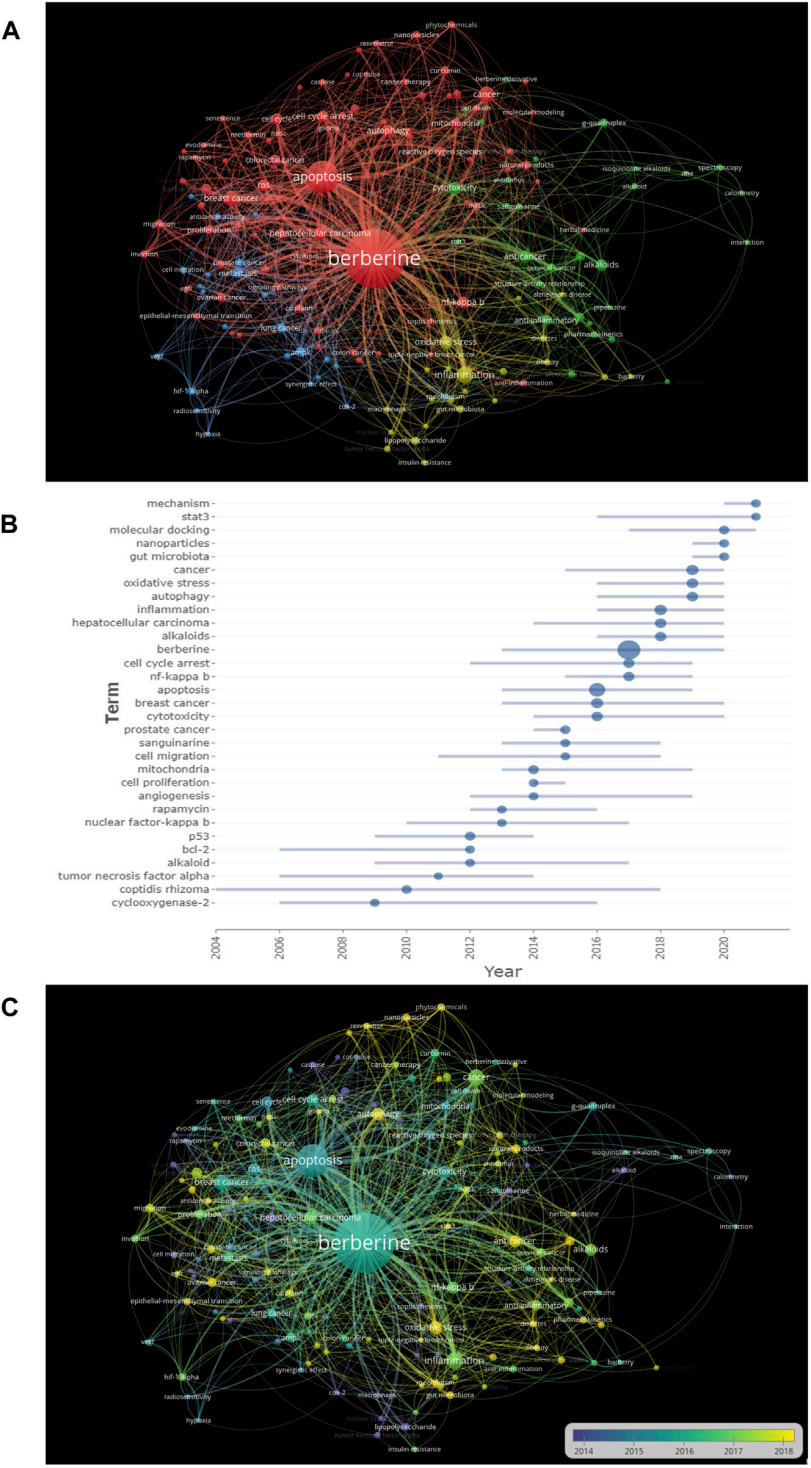
Analysis of keywords **(A)** the changing trend of research keywords from 2002 to 2021. **(B)** The evolution of keyword frequency. **(C)** The network map of keywords. Minimum number of occurrences of keywords ≥5.

Keywords are generally regarded as a significant index to reflect research frontiers and hotspots in a certain topic (Zhong And Song, 2008; Wu et al., 2021). We produced a map showing the co-occurrence of keywords using VOSviewer. The cooperation network (Figure 7A) clearly shows keywords changing from 2002 to 2021. At present, the keywords are main focused on “berberine”, “apoptosis” and “cancer”, and so on. Figure 7B shows the evolution of keywords in a typical publishing year. Initially,research on BBR mainly focused on “alkaloid”, and “tumor necrosis factor-alpha”; then, the research keywords shifted towards “prostate cancer”, “rapamycin” and “p53”; finally, the most recent keywords have become more diverse, with topics including “mechanism”, “cancer” and “gut microbiota”, and so on. Figure 7C shows the frequency of keywords in different time periods, the larger the size of the node, the higher the search frequency. In conclusion, “apoptosis”, “non-small cell lung cancer”, and “gut microbiota” were the most frequently searched terms.

## 4 Discussion

Chemotherapy and radiation therapy are standard treatments for patients with cancer. However, the resistance of malignant tumors to these treatments and the occurrence of major organ damage severely restrict the clinical outcomes of this disease (35). Moreover, current cancer treatments are inefficient, and the surgical prognosis is poor because tumor cells can invade and spread after treatment with a variety of chemical medications (Colagiuri et al., 2013). Natural herbal nutraceuticals have recently gained increased attention among popular clinical medications owing to their safety, adaptability to overcome therapeutic resistance, potential to reduce the adverse side effects of cancer treatments, low cost, and wide availability (Choudhari et al., 2019). Natural remedies can not only prevent drug resistance (38) but also have anti-tumor effects through various signaling pathways (Kumar and Adki, 2018; Al-Bari et al., 2021; Hashem et al., 2022). The prognosis of patients with malignant tumors can be improved by combining Chinese herbal therapies with other therapies (Mignani et al., 2018; Rejhová et al., 2018). BBR is a multi-target Chinese medicine monomer compound that not only regulates the growth of cancer cells but also acts as a combination chemotherapy drug for the treatment of tumors.

In this study, we conducted a bibliometric analysis to evaluate the hotspots and development trends of research on disciplines connected to BBR and tumors. We analyzed a total of 1,368 studies published from January 1, 2001, to December 31, 2021, and found that research on BBR and tumors has become increasingly frequent, suggesting that BBR may be a natural medication for treating tumors. China had the most relevant publications in the last 20 years, but the average number of citations per article was only 30.14%, significantly lower than that of other nations in terms of publications, suggesting that articles from China still have room for improvement. Among the top 10 countries with the greatest number of articles, China engages the most in international collaborations, primarily with the United States. China Medical University (Taiwan) published the most studies related to BBR and tumors worldwide. However, the University of Hong Kong ranked first in terms of average citations per article. Y. B. Feng, an author based in China, was the most productive and had the highest total citations. The journal that published the most articles was *Frontiers in Pharmacology* (29 articles), followed by *Molecules* (28 articles). We also analyzed the reference with the strongest citation burst; the latest literature of the co-cited reference was “Berberine, an epiphany against cancer,” which was published in *Molecules* in 2014. This article summarizes the molecular targets of BBR, several mechanisms by which BBR inhibits cancer, and the potential discovery of BBR derivatives for anti-cancer drugs (Ortiz et al., 2014). Our analysis of cooperation between countries, institutes, and institutes, showed that academic cooperation can promote the development of clinical and academic research.

### 4.1 Mechanism of berberine on cancers

As shown in Figure 6B, BBR has been used in the treatment of lung cancer (#13), prostate cancer (#6), and non-small-cell lung cancer, and has been a research hotspot in recent years (Figure 6C). The mechanisms of action of BBR in cancer have also been studied and mainly consist of inhibiting migration and invasion, inducing apoptosis, arresting the cell cycle, inhibiting proliferation, and promoting autophagy in tumors.

#### 4.1.1 Berberine can inhibit the migration and invasion of tumors

In clinical practice, most patients experience the invasion and migration of cancer cells from a single focus to other distant tissues. In 2015, research on migration and invasion became a hot topic (Figure 7B). A wide variety of migratory and invasion mechanisms are involved in cancer (Figure 6C). In triple-negative breast cancer cells, BBR reduces interleukin-8 (IL-8) expression by blocking the EGFR/MEK/ERK signaling pathway to prevent cell invasion and metastasis (Kim et al., 2018). BBR also affected cell migration and invasion in the human hepatoma HepG2 cell line by inhibiting the transforming growth factor (TGF-beta/Smad) signaling pathway (Du et al., 2021). Understanding cellular and molecular changes in these various migration/invasion programs will enable us to develop novel therapeutic approaches and provide insights into the spread of cancer cells.

#### 4.1.2 Berberine can promote tumor cell apoptosis

Apoptosis was the main keyword in BBR-related research (Figure 7A) and the median time of the research boom occurred around 2016 (Figure 7C). Apoptosis is an evolutionarily conserved cell death system responsible for eliminating cells during embryonic development and maintaining organismal homeostasis (Singh et al., 2019). The pathways of apoptosis are divided into the exogenous pathway, endogenous pathway, caspase cascade reaction, and caspase effects. Many biochemical processes, such as a loss of mitochondrial membrane potential, release of cytochrome C into the cytoplasm, expression of Bcl-2 family and caspase family proteins, and cleavage of poly ADP ribose polymerase, induced apoptosis in the skin squamous cell carcinoma A431 cell line after treatment with BBR (Wartenberg et al., 2003). Moreover, BBR induced apoptosis involved in the caspase-related mitochondrial pathway compared to normal liver HL-7702 cells, which was mediated by adenosine monophosphate-activated protein kinase (AMPK) and reduced the survival of human hepatoma HepG2, SMMC-7721, and Bel-7402 cells (Mitani et al., 2001). To reach the goal of treatment, researchers are currently modeling small molecules with apoptosis-promoting protein activity, which renders cells susceptible to mitochondrial apoptosis and triggers apoptosis.

#### 4.1.3 Berberine can block the cell cycle of tumor cells

Research on the effect of BBR on cell cycle arrest started around 2012 but was mainly concentrated in 2017 (Figure 7B). To some degree, changes in the cell cycle can either slow or accelerate cancer development. Previous studies demonstrated that BBR inhibits the growth of certain tumor cells by regulating their cell cycle. A study on the mechanism of BBR in breast cancer showed that the alkaloid BBR prevented breast cancer cells from entering the S phase and increased cancer cell sensitivity to treatment (Kim et al., 2010). In a human chondrosarcoma cell HBT-94 model, BBR activated the PI3K/Akt and p38 signaling pathways, increased the protein levels of p53 and p21, and triggered G2/M phase arrest, thus demonstrating anti-cancer effects (Eo et al., 2014). Gene stability is maintained by cell cycle arrest and cancer formation is significantly influenced by the incidence of cell cycle-regulated gene alterations. If DNA is damaged during a healthy cell cycle, the cell cycle stalls at a relevant checkpoint. Cell cycle arrest gives cells more time to repair damage, which lowers the likelihood of mutations and prevents tumor development. Therefore, targeting cell cycle checkpoints may significantly enhance cancer treatment.

#### 4.1.4 Berberine can inhibit tumor proliferation

Numerous studies have reported that BBR regulates cell signal transduction by inhibiting tumor cell proliferation. As shown in Figure 7B, the keyword “tumor proliferation” mainly appeared in 2014. BBR inhibited the growth of human colon cancer cell lines Caco-2 and Lovo by reducing citrate synthase activity (Mantena et al., 2006). Moreover, BBR inhibited melanoma by increasing miRNA-582-5p and miRNA-188-5p levels and decreasing the expression of cell cycle-related proteins in melanoma A375 cells (Lin et al., 2005). Research on the mechanism associated with tumor proliferation mainly focuses on material metabolism; therefore, a deeper understanding of the molecular connections between cellular metabolism and growth regulation may ultimately result in more effective cancer treatments.

#### 4.1.5 Berberine can induce autophagy of tumor cells

The cooperation network shown in Figure 7A shows that the keyword “autophagy” is closely connected with BBR. Autophagy, a type of programmed cell death, is crucial for preserving intracellular homeostasis. BBR induces autophagy by inhibiting the ERK1/2 signaling pathway in glioblastoma and further reduces temozolomide resistance (Qu et al., 2020). By inducing cytostatic autophagy and regulating the MAPK/mTOR/p70S6K and Akt signaling pathways, BBR inhibits the development of human gastric cancer cells both *in vitro* and *in vivo* (Zhang et al., 2020a). Despite substantial research on the regulation of autophagy by BBR in recent years, the precise mechanism remains unclear.

### 4.2 Applications of berberine

Figure 6 and Figure 7 list several conditions that can be regulated by BBR, including non-small-cell lung cancer, breast cancer, colorectal cancer, gut microbiota, and photodynamic therapy. Among these, the application of BBR in cancer has been the focus of research in recent years. The results are summarized in Table 8.

**TABLE 8:** BBR applications.

Application	Cell line	Effect	Reference
Non-small-cell lung cancer	A549, PC9, H1650, and H1299	Activation of the p38α MAPK signaling pathway	Wu et al. (2021)
		Induction of the protein expression of p53 and FOXO3a	
		Inhibit proliferation and induce apoptosis	
	A549, H157, H358, H460, H1299, H1975, and Lewis cells	Enhance tumor-infiltrating T-cell immunity and attenuate the activation of MDSCs and Tregs	Yang et al. (2021)
		Trigger PD-L1 degradation through the ubiquitin (Ub)/proteasome-dependent pathway	
	A549, NCI-H1299, and BEAS-2B	Suppression of epithelial–mesenchymal transition	Yin et al. (2022)
		Trigger cell cycle arrest	
		Suppression of HIF-1α expression	
	HCC827/AR, HCC827/AR0.5, HCC827/AR2, HCC827/ER, PC-9/AR, and PC-9/GR/AR	Act as a naturally existing MET inhibitor	Yu et al. (2020)
		Enhance induction of apoptosis through Bim elevation and Mcl-1 reduction	
Breast cancer	HEK293, SMCC-7721, and ZR-75-30	Decrease the expression of ephrin-B2 and its PDZ-binding proteins	Zhang et al. (2021a)
		Downregulate the phosphorylation of VEGFR2 and downstream signaling members (AKT and Erk1/2)	
		Downregulate the expression of MMP-2 and MMP-9	
	MCF-7 and MCF-7/MDR	Enhance sensitivity in drug-resistance breast cancer cells	Zhang et al. (2020a), Zhang et al. (2021b)
		Induce apoptosis	
	MDA-MB-231 and BT549	Induce DSB	Zhang et al. (2020b)
		Increase the release of cytochrome c	
		Trigger the caspase-9-dependent apoptosis	
Colorectal cancer	HT-29, SW480, and NIH3T3-Light2 cell lines	Suppression of the paracrine sonic hedgehog (SHH) signaling	Zhang et al. (2020c)
		Inhibition of the secretion and expression of SHH protein	
	HCT116 and SW480	Inhibit proliferation and induce G0/G1 phase arrest	Zhao et al. (2017)
		Knockdown of IGF2BP3 could suppress the PI3K/AKT pathway to inhibit cell proliferation and cycle transition	
	HCT-8, HCT-116, and HT-29	Suppress lipogenesis *via* promotion of PLZF-mediated SCAP ubiquitination	Zhao et al. (2022)
	HCT116, SW48, RKO, Caco-2, SW480, and HT-29	Suppression of DNA replication	Zheng et al. (2014)
Gut microbiota	Brain neuron cells	Accelerate the production of L-DOPA by intestinal bacteria	Zheng et al. (2021)
	*P. mirabilis*, *S*. *boydii*, and *B. fragilis* (intestinal bacterial strains)	Reduce the biosynthesis of TMAO by interacting with the enzyme/coenzyme containing (CutC) and (FMO)	Zhong and Song (2008)
	β-cells	Inhibit the biotransformation of DCA by *Ruminococcus bromii*	Zhuang et al. (2018)
Photodynamic therapy	ACHN, 786-O, and HK-2	Increase reactive oxygen species	[71]
		Increase autophagy levels and apoptosis by caspase 3 activity	
	A375, M8, SK-Mel-19, and the cisplatin-resistant cell lines A375/DDP, M8/DDP, and SK-Mel-19/DDP	Activate the P38 MAPK signaling pathway	[72]

#### 4.2.1 Non-small-cell lung cancer

Figure 6C shows a research hotspot focused on non-small cell lung cancer. Several mechanisms by which BBR inhibits non-small cell lung cancer have been reported. For example, Zheng et al. (2014) reported that BBR induces apoptosis of NSCLC cells to prevent growth and is involved in activating the p38α MAPK signaling pathway and subsequent increased protein expression of p53 and FOXO3a. BBR plays also an anti-tumor role from the perspective of immunity, as it can specifically bind to glutamic acid 76 of constitutive photomorphogenic-9 signalosome 5 (CSN5) and inhibit the PD-1/PD-L1 axis through its deubiquitylation activity, leading to the PD-L1 ubiquitination and destruction (Liu et al., 2020). In addition, BBR and its derivatives are considered potential drugs for the treatment of NSCLC. Liu et al. (2021) demonstrated that the derivative of BBR, demethyleneberberine (DMB), exerts an anti-tumor effect leading to cell arrest and cellular senescence in NSCLC. In addition, BBR can be combined with other drugs such as osimertinib (Chen et al., 2022). These findings demonstrate that BBR exerts anti-tumor effects through various mechanisms.

#### 4.2.2 Breast cancer

According to the keyword analysis (Figure 7A), “breast cancer” is also an aspect of BBR research. Ma et al. (2017) reported that BBR prevents the proliferation and migration of breast cancer ZR-75-30 cells by regulating ephrin-B2. BBR also increases chemosensitivity, reverses hypoxia-induced chemoresistance, and further induces apoptosis in breast cancer (Pan et al., 2017b; Pan et al., 2017c). Moreover, Zhao et al. (2017) reported that BBR inhibits triple-negative breast cancer. BBR induces caspase-9/cytochrome c-mediated apoptosis both *in vitro* and *in vivo* to inhibit the proliferation of TNBC cells. Thus, BBR has been used to treat breast cancer.

#### 4.2.3 Colorectal cancer

BBR inhibits colorectal tumor development A previous study showed that BBR reduces paracrine sonic hedgehog (SHH) signaling, which in turn reduces colon cancer growth *in vitro* and *in vivo* (Shen et al., 2021). This revealed a novel molecular mechanism for the anti-cancer effects of BBR. BBR inhibits proliferation through cell cycle arrest-related pathways. BBR has been reported to induce G0/G1 phase arrest in colorectal cancer cells by downregulating the targeted gene *IGF2BP3* (Zhang et al., 2020c). Moreover, BBR showed potential anti-migration and anti-invasion properties in cell lines including HCT-8, HCT-116, and HT-29. BBR reduces lipogenesis and the spread of colon cancer cells by promoting PLZF-mediated SCAP ubiquitination (Liu et al., 2022b). Meanwhile, several studies have reported on BBR combined with other drugs, for example, the combination with *Andrographis* to treat colorectal cancer (Zhao et al., 2022). BBR is well known as a potential drug for the treatment of colon cancer.

#### 4.2.4 Gut microbiota

Figure 6C shows that cluster 11 (gut microbiota) is a current research hotspot. The gut microbiota includes a large number and a wide range of species that are interdependent and interact with the host. The occurrence, development, and prognosis of many human diseases are closely related to intestinal flora. In 2021, Wang et al. (2021b) introduced oral BBR to accelerate the production of L-dopa by intestinal bacteria. The L-DOPA produced by intestinal bacteria enters the brain through circulation and is converted to dopamine, significantly increasing the brain dopamine levels of mice and improving PD expression. Ma et al. (2022) demonstrated that oral BBR reduced the biosynthesis of trimethylamine-N-oxide (TMAO), an atherogenic metabolite derived from the gut microbiota in the intestine, by interacting with the enzyme/coenzyme containing choline trimethylamine lyase (CutC) and lutein monooxygenase (FMO) in the intestinal microbiota, thus playing a role in the treatment of atherosclerosis. In addition, the human intestinal microbiome is a promising target for the treatment of type 2 diabetes. BBR reduces blood sugar levels by inhibiting the biotransformation of DCA by *Ruminococcus bromii* (Zhang et al., 2020b). Intestinal microorganisms affect the occurrence and development of metabolic diseases by regulating the metabolism of sugars, lipids, and amino acids and are inextricably linked with diseases of the neuropsychiatric, cardiovascular, urinary, and other systems. Therefore, it is of great significance to study the correlation between intestinal flora and diseases for the prevention and treatment of diseases and the maintenance of human health.

#### 4.2.5 Photodynamic therapy

BBR is well known for its anti-inflammatory, antioxidant, anti-diabetes, anti-obesity, and anti-cancer properties; however, little is known about its photosensitive properties, and it might serve as a new kind of photodynamic therapeutic agent. Our analysis of co-cited references (Figure 6C) showed that photodynamic therapy is a hotspot of current research in BBR. For example, a study that assessed the effects of BBR on PDT in renal cancer cell lines reported increased reactive oxygen species (ROS) levels after treatment with BBR associated with PDT, which was accompanied by increased autophagy levels and apoptosis due to caspase 3 activity (Lopes et al., 2020). Wang et al. (2021a) reported that a combination of cisplatin and BBR-PDT played a role in cisplatin-resistant melanoma cells. The experimental findings showed that mitochondrial apoptotic pathways that depend on caspases were the mode of cell death and that BBR photodynamic therapy modulated apoptosis by activating the P38 MAPK signaling pathway. The number of articles on BBR photosensitivity therapy is currently small, and most have focused on the treatment and application of cancer, which may suggest that photosensitivity therapy could provide a new method for cancer treatment.

BBR is a natural compound with great biological activity that is effective against various diseases. The literature review showed that increasing numbers of BBR derivatives have been created and used in disease research. BBR can be used as a combination drug in the study of drug-resistant cell lines and has shown significant effects. The ability of BBR to disrupt intracellular pathways and its intrinsic features has been the subject of numerous investigations in recent years. More importantly, BBR has been investigated as a curative medication in both animal models and human cell lines. However, there remain many issues to resolve; for example, BBR alone has not been tested in humans and it has weak water solubility, reduced oral absorption, and low bioavailability. Therefore, future studies should focus on the clinical use of BBR.

## 5 Conclusion

This study analyzed publications, research topics, research hotspots, and development trends of research in the field of BBR and tumors through systematic bibliometric analysis. Chinese researchers have produced the most publications; however, articles published in the United States ranked first in terms of average citations per article. The most prolific universities are China Medical University (Taiwan) and Sun Yat-sen University. The terms “mechanism,” “molecular docking,” and “oxidative stress” are now popular study topics related to BBR in tumors.

## Data Availability

The original contributions presented in the study are included in the article/Supplementary Material; further inquiries can be directed to the corresponding authors.
